# Supplementary material for: The Systems Biology Research Tool: evolvable open-source software
Source: BMC Syst Biol. 2008 Jun 29;2:55. doi: 10.1186/1752-0509-2-55 (PMC2446383; doi:10.1186/1752-0509-2-55)
Supplement: Additional file 1 — SBRT Archive. An archive of the current version of the Systems Biology Research Tool. [file 1752-0509-2-55-S1.zip › sbrt-1.4.0/doc/developers_guide/api/sbrt/kernel/math/HashQuadExpr.html]

HashQuadExpr


---


|  |  |  |
| --- | --- | --- |
| **Overview** \| **Package** \| **Class** \| **Use** \| **Tree** \| **Deprecated** \| **Index** \| **Help** | |  |
| **PREV CLASS**   **NEXT CLASS** | **FRAMES**    **NO FRAMES**     **All Classes** |  |
| SUMMARY: NESTED \| FIELD \| CONSTR \| METHOD | DETAIL: FIELD \| CONSTR \| METHOD |  |


---


## sbrt.kernel.math Class HashQuadExpr<V>

```
java.lang.Object
  sbrt.kernel.math.AbstractPolynomial<V>
      sbrt.kernel.math.HashQuadExpr<V>
```

**Type Parameters:**: `V` - the variable type.

**All Implemented Interfaces:**: MathExpr<V>, Polynomial<V>, QuadraticExpr<V>

---

``` public abstract class HashQuadExpr<V> extends AbstractPolynomial<V> implements QuadraticExpr<V> ```

This class is under construction.

**Author:**
:   This class is being written and documented by Jeremiah Wright
    while in the Wagner lab.

---

**Nested Class Summary**

**Nested classes/interfaces inherited from interface sbrt.kernel.math.QuadraticExpr**

`QuadraticExpr.Term<V>`


**Constructor Summary**

`HashQuadExpr(double constant)`
  
          Constructs a new quadratic expression.


**Method Summary**

**Methods inherited from class sbrt.kernel.math.AbstractPolynomial**

`checkConstant, getConstant, hashCode, toString`

**Methods inherited from class java.lang.Object**

`clone, equals, finalize, getClass, notify, notifyAll, wait, wait, wait`

**Methods inherited from interface sbrt.kernel.math.Polynomial**

`equals, getConstant, getTermArray, hashCode, plugInNumbers, terms, toString`

**Methods inherited from interface sbrt.kernel.math.MathExpr**

`getVariables, hasAllVariables, hasVariable, hasVariables, variables`

**Constructor Detail**

### HashQuadExpr

```
public HashQuadExpr(double constant)
```

:   Constructs a new quadratic expression.

    **Parameters:**: `constant` - the constant.


---


|  |  |  |
| --- | --- | --- |
| **Overview** \| **Package** \| **Class** \| **Use** \| **Tree** \| **Deprecated** \| **Index** \| **Help** | |  |
| **PREV CLASS**   **NEXT CLASS** | **FRAMES**    **NO FRAMES**     **All Classes** |  |
| SUMMARY: NESTED \| FIELD \| CONSTR \| METHOD | DETAIL: FIELD \| CONSTR \| METHOD |  |


---
